# Ultrasound-Guided Selective Nerve Root Block for Surgical Planning in Multilevel Cervical Disc Disease: A Technical Report

**DOI:** 10.7759/cureus.105690

**Published:** 2026-03-23

**Authors:** King Hei Stanley Lam, Yonghyun Yoon, Daniel Chiung-Jui Su, Anwar Suhaimi, Teinny Suryadi, Abdallah El-Sayed Allam, Manal Hassanien

**Affiliations:** 1 Faculty of Medicine, The Chinese University of Hong Kong, New Territories, HKG; 2 Faculty of Medicine, The University of Hong Kong, Hong Kong, HKG; 3 The Board of Clinical Research, The Hong Kong Institute of Musculoskeletal Medicine, Kowloon, HKG; 4 Orthopedics, International Academy of Musculoskeletal Medicine, Hongkong, HKG; 5 Orthopedics, International Academy of Regenerative Medicine, Incheon, KOR; 6 Orthopedics, MSKUS, San Diego, USA; 7 Orthopedic Surgery, Hallym University Kangnam Sacred Heart Hospital, Seoul, KOR; 8 Orthopedic Surgery, Incheon Terminal Orthopedic Surgery Clinic, Incheon, KOR; 9 Physical Medicine and Rehabilitation, Chi Mei Medical Center, Tainan, TWN; 10 Rehabilitation Medicine, University Malaya Medical Centre, Kuala Lumpur, MYS; 11 Rehabilitation Medicine, University Malaya, Kuala Lumpur, MYS; 12 Physical Medicine and Rehabilitation, Synergy Clinic, Jakarta, IDN; 13 Physical Medicine and Rehabilitation, Hermina Podomoro Hospital, Jakarta, IDN; 14 Morphological Madrid Research Center (MoMaRC), UltraDissection Spain EchoTraining School, Madrid, ESP; 15 Physical Medicine, Rheumatology and Rehabilitation, Tanta University Faculty of Medicine, Tanta, EGY; 16 Rheumatology, Assiut University Hospital, Assiut, EGY

**Keywords:** cervical disc, cervical vertebrae, discogenic neck pain, diskectomy, interventional, nerve block, preoperative care, radiculopathy, ultrasonography, ultrasound-guided hydrodissection

## Abstract

Selecting the appropriate surgical level in patients with multilevel cervical disc disease remains challenging when relying solely on clinical examination and magnetic resonance imaging (MRI) findings. Fluoroscopy-guided selective nerve root blocks (SNRBs) carry risks of vascular injury from inadvertent intra-arterial injection. This technical report describes the technical aspects of ultrasound-guided SNRB for preoperative surgical-level localization and provides a reproducible protocol for clinical implementation.

The procedure utilizes a high-frequency (12 MHz) linear transducer with color/power Doppler capability. Transverse process morphology guides level identification: C7 exhibits a rudimentary anterior tubercle with a prominent posterior tubercle; C6 displays a sharp, prominent anterior tubercle; and C3-C5 demonstrate the characteristic "two-humped camel" sign. The vertebral artery is identified anterior to C7 and confirmed with Doppler. Using an in-plane posterolateral to anteromedial approach, a 25-gauge needle is advanced toward the oval hypoechoic nerve root between the anterior and posterior tubercles (or anterior to the posterior tubercle for C7). After negative aspiration, 1 mL of 1% lidocaine is injected. For multilevel assessment, sequential blocks are performed, with a minimum of four-hour intervals between injections, blocking from caudal to cephalad. A positive response is defined as ≥50% reduction in arm pain on the Visual Analog Scale at 30 minutes post-injection.

This technique was developed and validated during a randomized controlled trial (NCT05145530), whose clinical outcomes have been published separately. The present technical report focuses on the procedural aspects. Ultrasound guidance enabled consistent visualization of target nerve roots in all 30 intervention patients (72 blocks). Power Doppler identified radicular arteries adjacent to nerve roots in 25% of procedures, allowing trajectory adjustment to avoid vascular puncture. No procedure-related complications occurred. The sequential block protocol successfully identified symptomatic levels in all patients, demonstrating the feasibility of the technique to inform surgical planning.

Ultrasound-guided cervical SNRB is a safe, radiation-free technique that enables real-time visualization of nerve roots and vascular structures, thereby enhancing the safety profile compared to fluoroscopy-guided approaches. The detailed step-by-step technique described herein should enable other clinicians to incorporate this approach into practice for accurate preoperative level selection in patients with multilevel cervical disc disease.

## Introduction

Magnetic resonance imaging (MRI) remains the standard for evaluating cervical disc disease, yet its limitations in surgical planning for multilevel pathology are well-documented. Asymptomatic disc degeneration occurs in up to 60% of individuals, and in symptomatic patients, morphological abnormalities often correlate poorly with the actual pain generator [1,2]. This diagnostic uncertainty poses a particular challenge in multilevel disease, where MRI may demonstrate degenerative changes at multiple levels without identifying which specific nerve root is responsible for radicular symptoms [3]. Consequently, surgeons may perform multilevel fusions based on imaging alone, exposing patients to increased risks of pseudoarthrosis, adjacent segment degeneration, dysphagia, and vertebral artery injury without guarantee of symptomatic relief [4-6].

Selective nerve root block (SNRB) has emerged as a valuable diagnostic tool to bridge this gap between imaging and symptomatology. By temporarily anesthetizing individual nerve roots, SNRB can confirm the pain generator and guide targeted surgical intervention [7]. However, the traditional fluoroscopy-guided approach carries inherent risks, including radiation exposure and potential catastrophic complications from inadvertent intra-arterial injection. Case reports have documented spinal cord infarction, brainstem herniation, cortical blindness, and death following cervical transforaminal injections, with the vertebral artery and radicular arteries being particularly vulnerable to needle injury or particulate steroid embolization [8-11]. While the precise incidence remains unknown, the severity of these complications has prompted a search for safer alternatives [12].

High-resolution ultrasound with color and power Doppler offers a technological solution. Modern ultrasound systems enable real-time visualization of cervical nerve roots, the vertebral artery throughout its course, and radicular arteries within the intervertebral foramen [13,14]. This direct visualization facilitates precise needle placement while avoiding critical vascular structures, eliminates ionizing radiation, and can be performed at the point of care [15]. Despite these advantages, standardized technical descriptions of ultrasound-guided cervical SNRB for preoperative surgical planning remain lacking in the literature.

Therefore, this technical report aims to (1) provide a comprehensive step-by-step description of ultrasound-guided cervical SNRB for surgical-level localization in multilevel cervical disc disease, (2) detail a sequential block protocol for identifying symptomatic levels, and (3) present safety and feasibility data from its implementation in a randomized controlled trial [16]. By providing this detailed guidance, we aim to facilitate broader adoption of this radiation-free alternative for preoperative surgical planning.

## Technical report

Methods (technical description)

Study Design and Setting

This technical report describes the ultrasound-guided SNRB procedure as performed during a randomized controlled trial (NCT05145530) conducted at a single academic center. The trial's clinical outcomes have been published separately [16]. The present report focuses exclusively on the technical aspects of the procedure to provide a reproducible guide for clinicians.

Equipment

Table 1 summarizes the recommended equipment for ultrasound-guided cervical SNRB. While this represents the authors' preferred configuration, equivalent alternatives are acceptable.

**Table 1:** Recommended equipment for ultrasound-guided cervical selective nerve root block NB: Essential ultrasound capabilities include B-mode imaging for nerve root identification, color Doppler for vascular mapping, and power Doppler for detecting low-velocity flow in small radicular arteries.

Category	Specifications
Ultrasound system	High resolution with linear array transducer (10-12 MHz); color and power Doppler capability
Needle	25-gauge, 50-mm (2-inch) echogenic tip needle (e.g., Pajunk ( Geisingen, Germany )) or standard hypodermic needle
Injectate	1% lidocaine hydrochloride, preservative-free (1 mL per level)
Supplies	Sterile transducer cover, antiseptic solution (chlorhexidine 2% in 70% alcohol), sterile gloves, drapes, gauze
Syringes	3-mL Luer lock syringes

Patient Positioning

The patient is positioned supine with the neck in neutral to slight extension. A small rolled towel beneath the upper thoracic spine facilitates gentle extension, opening the intervertebral foramina and improving acoustic windows. The head is rotated 15-30 degrees contralateral to the symptomatic side to improve posterolateral neck access while maintaining patient comfort; excessive rotation (>30 degrees) should be avoided as it can distort normal anatomy. Arms are positioned comfortably at the sides. The ultrasound machine is placed contralateral to the operator, with the screen at eye level. The operator sits or stands at the head of the bed, facing the patient's affected side.

Pre-procedure Scanning and Level Identification

**Figure 1:**
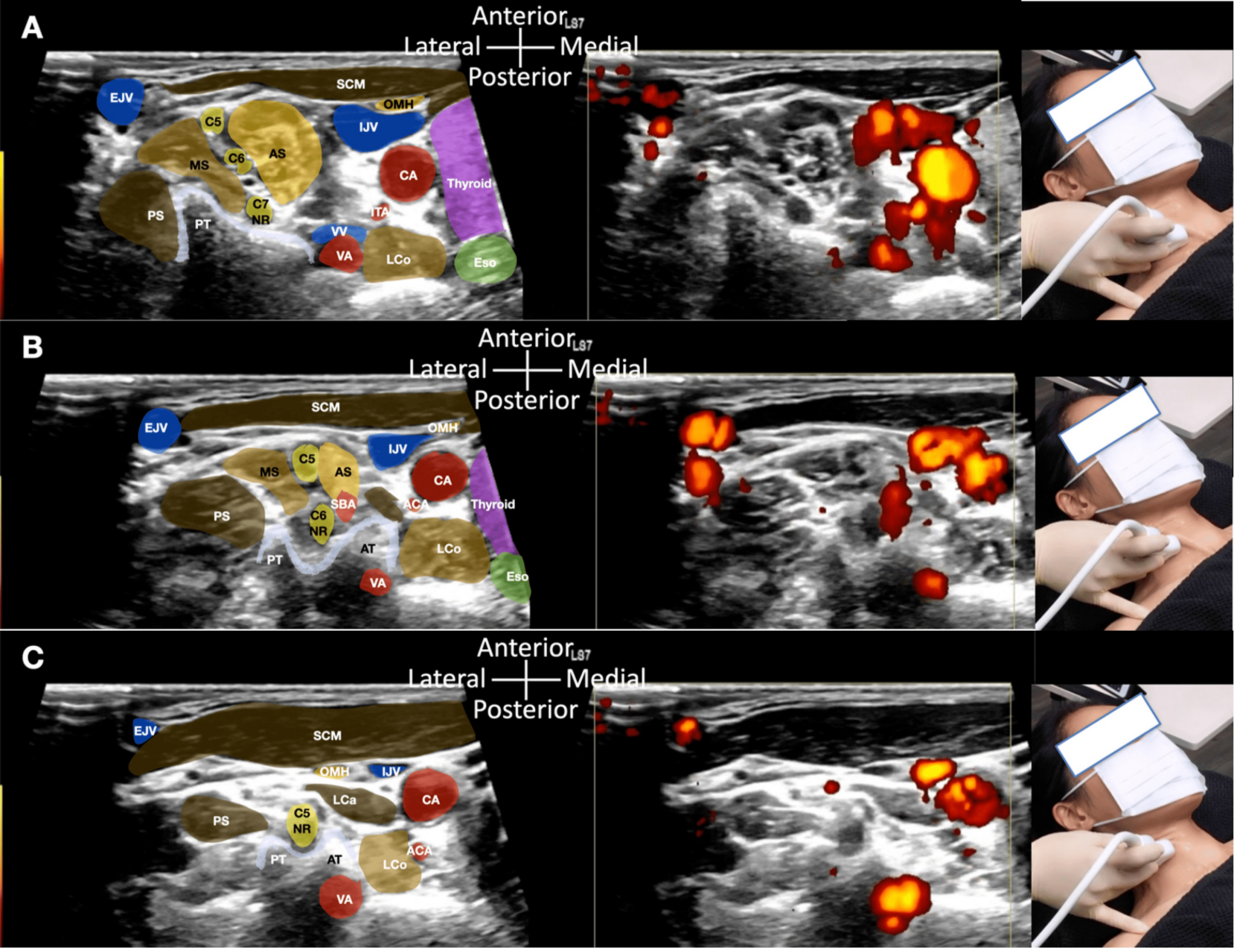
Ultrasound anatomy for cervical-level identification (A) Ultrasonogram showing the C7 transverse process, with rudimentary anterior tubercle and prominent posterior tubercle and C7 root, and the vertebral artery before entering the C6 foramen transversarium. (B) Ultrasonogram illustrating the C6 transverse process, with a sharp, prominent anterior tubercle and posterior tubercle with the C6 root in between them. (C) Ultrasonogram demonstrating the C5 transverse process, with the anterior tubercle and posterior tubercle forming a “two-humped camel” sign and the C5 root in between them. AS: anterior scalene muscle, AT: anterior tubercle, CA: carotid artery, EJV: external jugular vein, ESO: esophagus, IJV: internal jugular vein, ITA: inferior thyroid artery, LCa: longus capitis muscle, LCo: longus coli muscle, MS: middle scalene muscle, OMH: omohyoid muscle, PS: posterior scalene muscle, PT: posterior tubercle, SCM: sternocleidomastoid muscle, VA: vertebral artery, VV: vertebral vein.

Systematic-level identification begins with a transverse scanning approach, starting at the C7 anchor point and moving cephalad. Figure 1 illustrates the key ultrasound anatomical landmarks.

At the C7 level (anatomical anchor): The transducer is placed transversely over the lower lateral neck. C7 is identified by its unique morphology: a rudimentary anterior tubercle and prominent posterior tubercle, a "one-humped" appearance (Figure 1A). The vertebral artery is visualized anterior to the C7 transverse process before entering the C6 foramen transversarium; color Doppler confirms vascular identity. The C7 nerve root appears as an oval hypoechoic structure with a hyperechoic rim, located immediately anterior to the posterior tubercle.

At the C6 level: Sliding the transducer cephalad reveals a sharp, prominent anterior tubercle and well-defined posterior tubercle (Figure 1B). The key distinction from C7 is the well-developed anterior tubercle. The vertebral artery is now within the foramen transversarium, deep to the transverse process. The C6 nerve root lies between the anterior and posterior tubercles.

At the C5 level: Further cephalad movement reveals the characteristic "two-humped camel" sign, formed by equally prominent anterior and posterior tubercles (Figure 1C). The C5 nerve root is visualized between them.

At the C4 and C3 levels: The same "two-humped camel" morphology persists, though transverse processes become progressively smaller cranially.

Vascular mapping: Before needle insertion, a comprehensive color and power Doppler survey is performed at each target level. Power Doppler is preferred for its higher sensitivity to low-velocity flow in small vessels. The vertebral artery is traced from its C6 entry through the C5 and C4 foramina. Radicular arteries, small branches supplying nerve roots and the spinal cord, are identified when visible within the intervertebral foramen or adjacent to the target nerve root. This mapping identifies "no-go" zones and guides safe needle trajectory. If a radicular artery lies in the planned needle path, trajectory is adjusted; if avoidance is impossible, the block is abandoned.

Needle Technique

Skin preparation and draping: After optimal transducer position is confirmed, the skin is marked at the planned insertion site. Strict aseptic technique is employed: surgical hand hygiene, sterile gloves and gown, skin preparation with antiseptic solution using concentric circular technique, sterile transducer cover with gel, and sterile drape over the procedural field.

Approach: An in-plane, posterolateral to anteromedial approach is used for all levels. This allows continuous visualization of the entire needle shaft from skin entry to target. The transducer is oriented transversely, and the needle is inserted from the lateral neck, directed medially toward the target nerve root.

Needle insertion: The transducer is held in the non-dominant hand, maintaining stable transverse orientation at the target level. A 25-gauge, 50-mm needle is inserted approximately 1-1.5 cm lateral to the lateral transducer border, with the bevel facing the ultrasound beam to enhance visualization. The needle is advanced slowly with gentle, steady pressure, maintaining alignment with the ultrasound beam. The needle tip appears as a bright echogenic dot, and the shaft as a linear hyperechoic structure. If visualization is poor, small transducer adjustments or slight needle movements can help confirm the location.

Target localization: For C5, C6, and cephalad levels, the ideal target is the triangular space between the anterior tubercle, posterior tubercle, and the nerve root itself, an extraforaminal position critical for safety. The needle tip is positioned immediately adjacent to the nerve root, within its surrounding fascial plane, but outside the intervertebral foramen to prevent unintended epidural or intrathecal spread. For C7, which lies anterior to the posterior tubercle, the target is the space immediately anterior to the posterior tubercle, again maintaining an extraforaminal position.

**Figure 2:**
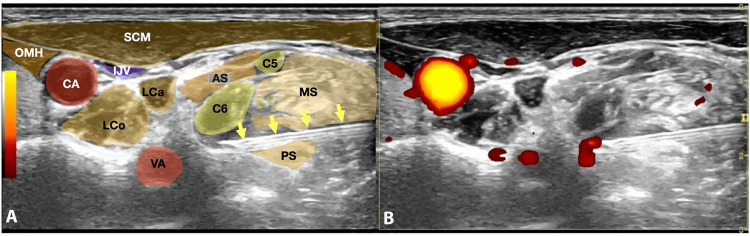
Ultrasound-guided C6 selective nerve root block: needle trajectory and target position (A) Ultrasound image with colored anatomical overlay at the C6 level, identifying key structures including the sternocleidomastoid muscle, scalene muscles, vertebral artery, carotid artery, and internal jugular vein. (B) Corresponding B-mode ultrasound image showing the needle trajectory and final needle tip position adjacent to the C6 nerve root, anterior to the posterior tubercle. Note the critical vascular structures, the vertebral artery (VA), carotid artery (CA), and internal jugular vein (IJV), that are identified and avoided. AS: anterior scalene muscle, CA: carotid artery, IJV: internal jugular vein, LCa: longus capitis muscle, LCo: longus colli muscle, MS: middle scalene muscle, OMH: omohyoid muscle, PS: posterior scalene muscle, SCM: sternocleidomastoid muscle, VA: vertebral artery.

Figure 2 illustrates the optimal needle trajectory and final needle position for a C6 SNRB, demonstrating the relationship between the needle tip, nerve root, and surrounding anatomical structures.

Confirmation of needle position: Once the needle tip is visualized adjacent to the nerve root, subtle adjustments may optimize position. The tip should move freely with the nerve root during gentle transducer pressure changes, indicating proper fascial plane placement, a "hydrodissection" or tissue plane confirmation (Video 1). Gentle injection of 0.1-0.2 mL of normal saline or lidocaine can confirm appropriate spread; the injectate should surround the nerve root without tracking medially toward the foramen or laterally into muscle planes. If the injectate is not visualized or spreads away, the needle position should be reassessed.

**Video 1:** Real-time ultrasound-guided C6 selective nerve root block using the hydrodissection technique This video demonstrates the complete procedural workflow for a right-sided C6 selective nerve root block using the hydrodissection technique. The transverse ultrasound view identifies the tallest C6 transverse process with its sharp anterior tubercle. A 25-gauge hypodermic needle is advanced using an in-plane, posterolateral to anteromedial approach. Using the hydrodissection technique, the needle tip is guided toward the floor of the extraforaminal space immediately adjacent to the C6 nerve root, between the nerve root and the posterior tubercle. Following negative aspiration, 1 mL of 1% lidocaine is injected slowly under real-time visualization, with the injectate observed as a hypoechoic fluid collection expanding around and surrounding the nerve root. Note the continuous visualization of the needle shaft and tip throughout the procedure, as well as the clear depiction of surrounding anatomical structures, including the vertebral artery (VA) deep to the transverse process.

Injection: After negative aspiration for at least five seconds (observed visually and under color Doppler to detect vascular flash), 1 mL of 1% lidocaine is injected slowly under real-time ultrasound visualization (approximately 0.2 mL/second). The injectate should appear as a hypoechoic or anechoic fluid collection expanding around the nerve root. Power Doppler can be activated during injection to immediately detect inadvertent intravascular spread, which would appear as turbulent color flow. If intravascular injection is suspected, the procedure is terminated immediately.

Post-injection observation: The needle is withdrawn, and gentle pressure is applied. The patient remains supine for 30 minutes for pain assessment and monitoring for adverse effects. Vital signs are monitored, and the patient is observed for signs of local anesthetic toxicity or vasovagal reactions.

Sequential Block Protocol for Multilevel Assessment

For patients with suspected multilevel involvement based on clinical presentation and MRI findings (typically showing ≥2 levels of disc degeneration, foraminal stenosis, or nerve root compression), a systematic sequential block protocol identifies the primary pain generator(s).

The general principles are as follows: (1) one level blocked at a time, (2) a minimum of four-hour intervals between blocks to avoid lingering anesthetic effects, (3) no analgesics within 12 hours of procedure, (4) pain intensity documented using the Visual Analog Scale (VAS, 0-10) [17] immediately before each block and at 30 minutes post-injection, and (5) provocative maneuvers (Spurling test, neck rotation, shoulder abduction) performed pre- and post-block to assess functional improvement

The following presents the block order algorithm.

Initial level selection: The more caudal level on the symptomatic side is blocked first (e.g., if MRI shows pathology at C5-C6 and C6-C7 with right-sided symptoms, the right C7 nerve root is blocked first). This caudal-to-cephalad approach prevents residual anesthetic from a cephalad block from confounding the interpretation of a more caudal level, as spread is more likely caudal.

Pain assessment at 30 minutes: Patient rates arm pain intensity using the VAS and reports percentage pain reduction compared to baseline. Patient performs previously painful movements to assess functional improvement.

Decision algorithm: (1) >80% pain relief: Blocked level confirmed as primary pain generator. Cephalad level is not blocked; surgical planning proceeds for single-level intervention. (2) 50%-80% pain relief: Partial response suggests possible contribution from cephalad level. The patient waits four hours, then the cephalad root is blocked.​​​​​​​ (3) <50% pain relief: Blocked level is not the primary pain generator. The patient waits four hours, then the cephalad root is blocked.

Second block interpretation: Following the four-hour interval, the cephalad root is blocked using the identical technique. Pain reassessed at 30 minutes. If ≥50% relief, that level is considered positive. Surgical planning includes all levels demonstrating ≥50% pain relief on sequential testing.

For three- or four-level disease: Blocks are performed over two consecutive days following the same sequential protocol. At day 1, the two most caudal levels are assessed at four-hour intervals. At day 2, remaining cephalad levels are assessed, always blocking from caudal to cephalad. The patient maintains a pain diary between sessions.

Documentation: A standardized block record documents baseline VAS, target level and side, ultrasound findings (vertebral artery position, radicular arteries visualized), needle approach and final tip position, injectate volume and resistance, VAS at 30 minutes, percentage pain reduction, and any adverse effects.

Interpretation Criteria and Surgical Planning

A positive block response is defined as ≥50% reduction in arm pain on VAS at 30 minutes following SNRB, accompanied by improvement in provocative maneuver testing. This threshold was selected based on literature demonstrating a correlation with successful surgical outcomes. Block results are documented in a sealed envelope and provided to the surgeon prior to the planned anterior cervical discectomy and fusion (ACDF). The surgeon is blinded to the block results until after completing standard preoperative assessment based on clinical examination and MRI. The surgical plan includes fusion at all levels with positive block responses, regardless of MRI degeneration grade. Levels not meeting the ≥50% threshold are excluded from the surgical plan, even if the MRI shows significant degenerative changes.

Safety Considerations and Troubleshooting

Vascular identification: If color Doppler reveals a radicular artery adjacent to the target nerve root that cannot be avoided by needle repositioning, the block is abandoned. Patient's safety supersedes procedural completion. Alternative approaches (oblique view, slight caudal-to-cephalad angulation) may be attempted, but safety remains paramount.

Intravascular injection: If real-time ultrasound during injection demonstrates turbulent flow on Doppler or the patient reports immediate dizziness, perioral numbness, or metallic taste, the procedure is terminated immediately. The patient is monitored for signs of local anesthetic systemic toxicity. Resuscitation equipment, including lipid emulsion, should be immediately available.

Patient discomfort: If paresthesia occurs during needle advancement, withdraw slightly and redirect. Persistent paresthesia prompts reassessment of needle position. Paresthesia during injection may indicate intraneural placement and warrants immediate cessation.

Inadequate visualization: In challenging anatomy (short neck, obesity, previous surgery), reduce transducer frequency to 8-10 MHz to improve penetration. Gentle compression can improve tissue contact and image quality. "Toeing" the transducer (angling so the scanning surface becomes more parallel to the needle trajectory) enhances needle visualization (Video 1). If adequate visualization cannot be achieved, the procedure should not be attempted.

Results

Patient and Procedure Characteristics

The ultrasound-guided SNRB technique was successfully completed in all 30 intervention patients, with no failed or abandoned procedures. A total of 72 nerve root blocks were performed (mean 2.4 blocks per patient, range 1-4). The standardized scanning protocol provided adequate visualization of target nerve roots in all cases, with the systematic C7-to-cephalad approach proving reliable regardless of body habitus or degenerative changes.

Technical Success and Image Quality

Adequate ultrasound visualization of target nerve roots was achieved in 100% (72/72) of blocks. The vertebral artery was successfully identified using color Doppler at C7 in all patients, providing an essential safety checkpoint before needle insertion. In one patient (3.3%) with a tortuous vertebral artery course, the vessel could not be confidently traced beyond C6, but target nerve roots remained clearly visible for safe block performance.

Power Doppler revealed small radicular arteries adjacent to the target nerve root in 18 of 72 procedures (25%, 95% CI: 15.6%-36.9%). These vessels were identified at the C5 (n = 4), C6 (n = 8), and C7 (n = 6). In all cases, the needle trajectory was adjusted to avoid these vessels, a capability not possible with fluoroscopy. In two cases (2.8%), the initial planned trajectory would have intersected a radicular artery; ultrasound guidance enabled identification of this risk and trajectory modification. No vascular punctures occurred.

Mean procedure time from transducer placement to needle withdrawal was 8.4 ± 2.3 minutes per level (range 5-14 minutes). All blocks were completed within a single outpatient visit, with the sequential protocol successfully executed within the prescribed four-hour intervals.

Block Protocol Implementation

The sequential block protocol was successfully implemented in all patients. The distribution of positive blocks per patient was as follows: single-level positive in 16 patients (53.3%), two-level positive in 10 patients (33.3%), and three-level positive in 4 patients (13.3%).

Among patients with single-level positive blocks, preoperative MRI had demonstrated two or more levels of degenerative change in 14 of 16 patients (87.5%), highlighting the ability of SNRB to identify a single pain generator despite multilevel imaging abnormalities. The complete distribution of block results and corresponding surgical levels has been published previously [16].

Pain Response to Diagnostic Blocks

Mean baseline VAS before blocks was 6.8 ± 1.4, decreasing to 2.1 ± 1.6 at 30 minutes post-injection. Pain relief distribution was >80% relief in 41 blocks (56.9%), 50%-80% relief in 24 blocks (33.3%), and <50% relief in 7 blocks (9.7%). Blocks achieving <50% relief were at levels subsequently excluded from surgery. The correlation between block responses and postoperative outcomes is detailed in the separate clinical publication [16].

Safety Outcomes

No immediate procedure-related complications occurred in any of the 72 nerve root blocks. Specifically, there were no instances of intravascular injection, hematoma, vasovagal reactions, local anesthetic toxicity, new neurological deficits, or allergic reactions. Three patients (10%) reported transient injection site discomfort that resolved within 24 hours without intervention.

Regarding postoperative adverse events, one patient in the SNRB group (3.3%) experienced transient dysphagia and hoarseness following four-level ACDF, a recognized complication of multilevel anterior cervical surgery, rather than the preoperative blocks. Symptoms resolved within four weeks. Postoperative complications in the control group (n = 2, 6.7%) are detailed in the separate trial publication [16].

Summary of Technical Validation

The ultrasound-guided SNRB technique proved technically feasible, with consistent target visualization (100%), reliable sequential block implementation, and zero procedure-related complications in 72 blocks. The ability to identify radicular arteries in 25% of procedures and adjust needle trajectory accordingly represents a key safety advantage over fluoroscopy-guided techniques. The mean procedure time of 8.4 minutes per level demonstrates efficient outpatient applicability. Clinical outcomes validating diagnostic accuracy have been reported separately [16].

## Discussion

Overview of findings

This technical report demonstrates that ultrasound-guided SNRB is feasible, safe, and can provide clinically actionable information for surgical-level selection in patients with multilevel cervical disc disease. Key technical findings include (1) consistent visualization of target nerve roots in 100% of procedures, (2) identification of radicular arteries in 25% of blocks with subsequent trajectory adjustment, (3) zero procedure-related complications in 72 blocks, and (4) successful implementation of a sequential block protocol that informed surgical planning in all patients. The detailed step-by-step technique described herein may enable other clinicians to incorporate this approach into their practice.

Potential advantages over fluoroscopy-guided techniques

The traditional fluoroscopy-guided approach to cervical SNRB, while effective, carries well-documented risks. The vertebral artery traverses the foramen transversarium from C6 to C1, placing it in close proximity to target nerve roots during transforaminal injections [18]. Case reports have documented catastrophic outcomes, including brainstem and spinal cord infarction, following inadvertent intra-arterial injection of particulate corticosteroids [11,19,20]. While the exact incidence of these complications remains unknown, their severity has prompted interest in safer alternatives [21].

Ultrasound guidance may offer several advantages that address these safety concerns. First, real-time visualization of vascular structures using color and power Doppler allows identification of the vertebral artery and radicular arteries before needle insertion. In our series, radicular arteries were identified adjacent to the target nerve root in 25% of procedures, enabling trajectory adjustment to avoid puncture, a capability not available with fluoroscopy.

Second, continuous needle tip visualization helps ensure the needle remains outside the intervertebral foramen and away from the vertebral artery. The extraforaminal target position between the nerve root and posterior tubercle may provide an additional margin of safety while maintaining block selectivity.

Third, the elimination of ionizing radiation benefits both patients and operators. While radiation from a single fluoroscopy-guided block may be low, cumulative occupational exposure carries documented health risks [22].

Fourth, real-time observation of injectate spread allows immediate detection of intravascular injection (visible as turbulent flow on Doppler) [23]. Narouze has noted that ultrasound "prevents" whereas contrast fluoroscopy only "detects" intravascular injection, a potentially important distinction [23].

Clinical validation and impact on surgical planning

The clinical utility of this technique is suggested by outcomes from our randomized controlled trial, reported separately [16]. As detailed in that publication, 53.3% of patients in the intervention group underwent single-level surgery despite MRI evidence of multilevel disease, a finding consistent with the known false-positive rate of cervical MRI in asymptomatic populations [24,25]. Among patients with single-level positive blocks, 87.5% had an MRI demonstrating two or more levels of degenerative change, highlighting the limited specificity of MRI alone for identifying pain generators.

The sequential block protocol, with its caudal-to-cephalad approach and four-hour inter-block interval, proved workable for identifying primary pain generators. The ≥50% pain reduction threshold at 30 minutes post-injection is consistent with established criteria for diagnostic block interpretation [26,27]. The observation that 9.7% of blocks achieved <50% relief and were subsequently excluded from surgery suggests the protocol may help prevent unnecessary multilevel fusion in some patients.

Technical considerations and learning curve

Successful performance of ultrasound-guided cervical SNRB requires familiarity with cervical sonography and relevant anatomy. Based on our experience, operators with prior ultrasound-guided procedure experience may require 10-20 supervised procedures to achieve consistent proficiency. The systematic level identification approach starting at C7, with its characteristic rudimentary anterior tubercle, provides a reliable anatomical anchor that minimizes the risk of level misidentification.

Several technical points warrant emphasis.

Transducer Selection

A high-frequency linear transducer (10-12 MHz) provides optimal resolution; power Doppler is more sensitive than color Doppler for detecting low-velocity flow in small radicular arteries [28].

Needle Selection

A 25-gauge, 50-mm needle offers balanced visibility and patient comfort; echogenic tip needles enhance visualization, particularly with steep angles or deeper anatomy [29].

Injectate Volume

The 1 mL volume used in this protocol is smaller than volumes typically reported for fluoroscopic blocks (1.5-2.5 mL) [30]. Won et al. demonstrated that 1 mL volumes consistently spread to the dorsal root ganglion without epidural extension [31].

Patient Positioning

Supine position with slight contralateral rotation (≤30 degrees) optimizes access while avoiding distortion of critical anatomical relationships.

Relationship to prior work

Anderberg et al., using fluoroscopy-guided SNRB in 30 patients with multilevel cervical disease, reported that block results correlated poorly with both MRI findings and neurological examination [3]. Our findings are consistent with their work in demonstrating that SNRB can identify pain generators not apparent on MRI or clinical examination. The present report extends this foundational work by describing an ultrasound-guided approach that eliminates radiation exposure and enables real-time vascular visualization.

Jee et al. compared ultrasound-guided versus fluoroscopy-guided cervical SNRB for therapeutic purposes in 46 patients, finding no significant difference in pain reduction but significantly shorter procedure times and no radiation exposure in the ultrasound group [13]. Our mean procedure time of 8.4 minutes per level compares favorably with their reported times.

Won et al. investigated injectate spread patterns in ultrasound-guided cervical nerve root blocks, finding that 1 mL volumes consistently spread to the dorsal root ganglion without epidural extension [31]. Our finding that 90.3% of blocks achieved ≥50% pain relief supports the diagnostic adequacy of this low-volume approach.

Safety advantages of ultrasound guidance

The absence of procedure-related complications in this series (0% in 72 blocks) is encouraging, though the 95% CI (0%-5%) suggests the true complication rate could be as high as 5%. This compares favorably with reported rates for fluoroscopy-guided techniques, though direct comparison is limited by differing study populations and methodologies [11].

The safety profile of ultrasound-guided cervical injections may relate to several factors: avoidance of vascular structures identified on pre-procedural Doppler, continuous needle tip visualization, extraforaminal needle placement, and real-time observation of injectate spread. Additionally, the use of non-particulate local anesthetic (lidocaine) for diagnostic blocks eliminates the risk of particulate embolization should intravascular injection inadvertently occur.

An additional technical consideration is the choice of coupling medium. To avoid introducing gel fragments into the needle tract, which can induce perineural inflammation [32], we utilize antiseptic solution (chlorhexidine in alcohol) as the coupling medium between the sterile transducer cover and skin. This practice aligns with principles of nerve hydrodissection, where the goal is to use injectate to gently separate the nerve from surrounding tissues without extraneous irritants [33,34].

One patient in the SNRB group experienced transient postoperative dysphagia and hoarseness following four-level ACDF, a recognized complication of multilevel anterior cervical surgery attributed to retraction-related recurrent laryngeal nerve neuropraxia rather than preoperative blocks [35]. This highlights the morbidity associated with extensive surgery and underscores the importance of accurate preoperative level selection to minimize fusion extent when possible.

Limitations

This technical report has several limitations. First, the procedure is operator-dependent and requires specific training; our results reflect the experience of operators with expertise in interventional musculoskeletal ultrasound and may not be immediately generalizable. Based on extrapolation from other ultrasound-guided procedures, 20-30 supervised procedures may be necessary for competence [36].

Second, anatomic variations may complicate the procedure. In one patient (3.3%), a tortuous vertebral artery could not be confidently traced beyond the C6, though this did not prevent successful block performance. Operators should be prepared to abandon the procedure if anatomy precludes safe access.

Third, the sequential block protocol requires patients to remain in the procedure area for several hours, which may be impractical in some settings. The four-hour interval is conservative; future studies may determine whether shorter intervals provide equivalent diagnostic accuracy.

Fourth, this single-center study conducted in Egypt may limit generalizability to other populations and healthcare settings, though the anatomical landmarks described are universal. The sample size of 30 patients (72 blocks), while adequate to demonstrate technical feasibility, is relatively small. The 95% CI for the complication rate (0%-5%) suggests the true rate could be as high as 5%, and larger multicenter studies are warranted.

Finally, this report focuses on diagnostic applications; therapeutic applications (e.g., corticosteroid or dextrose injections) require separate investigation with additional safety considerations.

Future directions

Several avenues for future research merit consideration: (1) comparative effectiveness studies directly comparing ultrasound-guided versus fluoroscopy-guided SNRB for preoperative surgical planning, including formal cost-effectiveness analyses; (2) optimization of the sequential block protocol (e.g., refining inter-block intervals) and investigation of adjunctive technologies (e.g., machine learning, contrast-enhanced ultrasound, fusion imaging) to streamline evaluation and identify patients most likely to benefit; (3) standardization of training and competency assessment through simulation-based modules and validated checklists to facilitate wider adoption [36]; and (4) long-term outcomes following surgery guided by ultrasound SNRB (1, 2, and 5 years) to assess sustainability of benefits and potential reduction in adjacent segment degeneration, as well as therapeutic applications of ultrasound-guided cervical nerve root injections in non-surgical candidates [37].

## Conclusions

This technical report demonstrates that ultrasound-guided SNRB is a feasible technique for preoperative surgical-level localization in patients with multilevel cervical disc disease. In this series of 72 blocks, the procedure enabled real-time visualization of target nerve roots and identification of radicular arteries in 25% of cases, a capability not available with fluoroscopy-guided techniques, with no procedure-related complications. These findings suggest potential safety advantages, though they derive from a single-center study with a relatively small sample size. The detailed step-by-step technique described herein may assist other clinicians in evaluating this radiation-free approach in their own practice. Further multicenter studies with long-term follow-up are warranted to confirm these findings and establish the generalizability of this technique.
